# Supplementary material for: Oxidative Stress Induces a VEGF Autocrine Loop in the Retina: Relevance for Diabetic Retinopathy
Source: Cells. 2020 Jun 11;9(6):1452. doi: 10.3390/cells9061452 (PMC7349409; doi:10.3390/cells9061452)
Supplement: Supplementary file 1 [file cells-09-01452-s001.pdf]

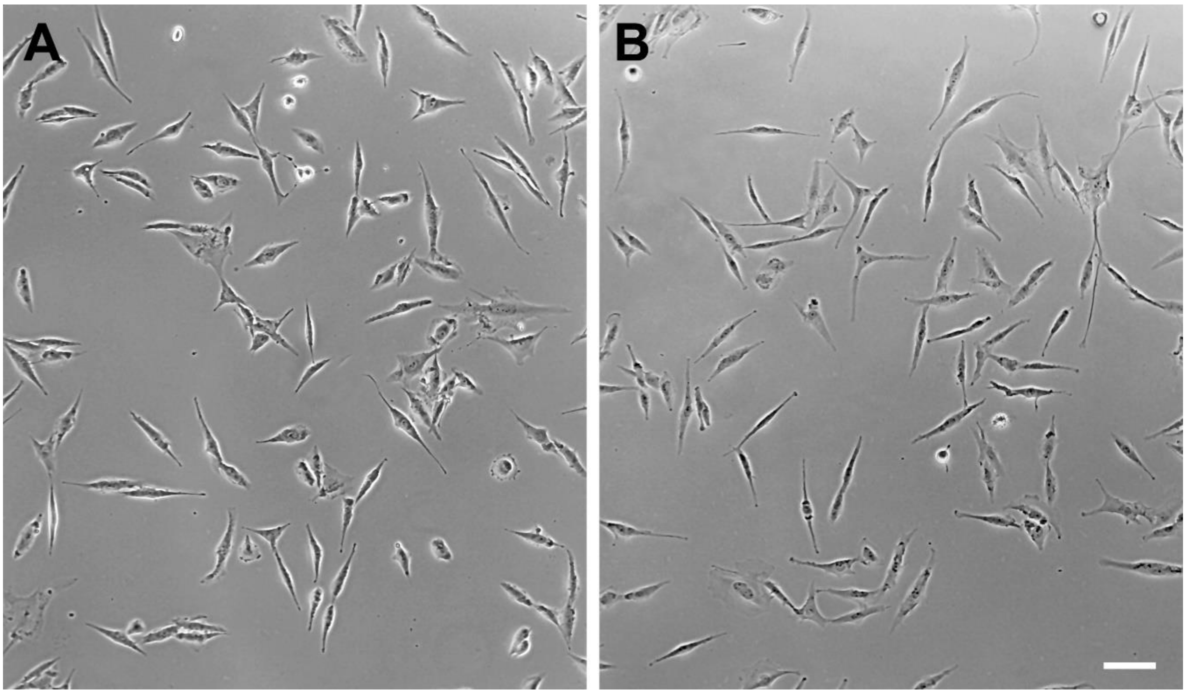

Figure S1. Morphology of living MIO-M1 cells (**A**) and of MIO-M1 cells after fixation for 20 min in 4% paraformaldehyde in PBS and permeabilization with 0.3% Triton X-100 in PBS for 5 min (**B**). DIC microscopy. Scale bar, 100 µm.

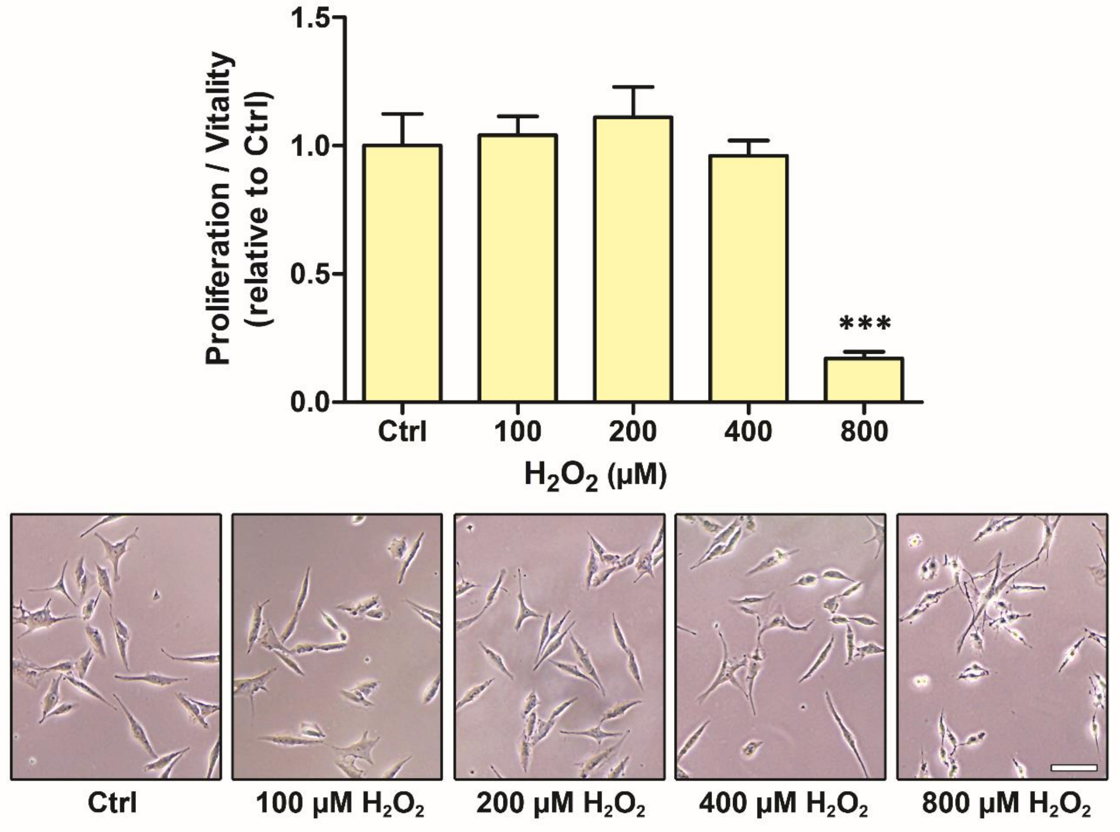

Figure S2. Proliferation/vitality of MIO-M1 cells treated with different doses of H_2O_2 to determine the H_2O_2 concentration to be used in the experiments with MIO-M1 cells. *** $p < 0.001$ relative to controls (Ctrl). $n = 4$. The photomicrographs were taken with DIC microscopy. Scale bar, 100 µm.

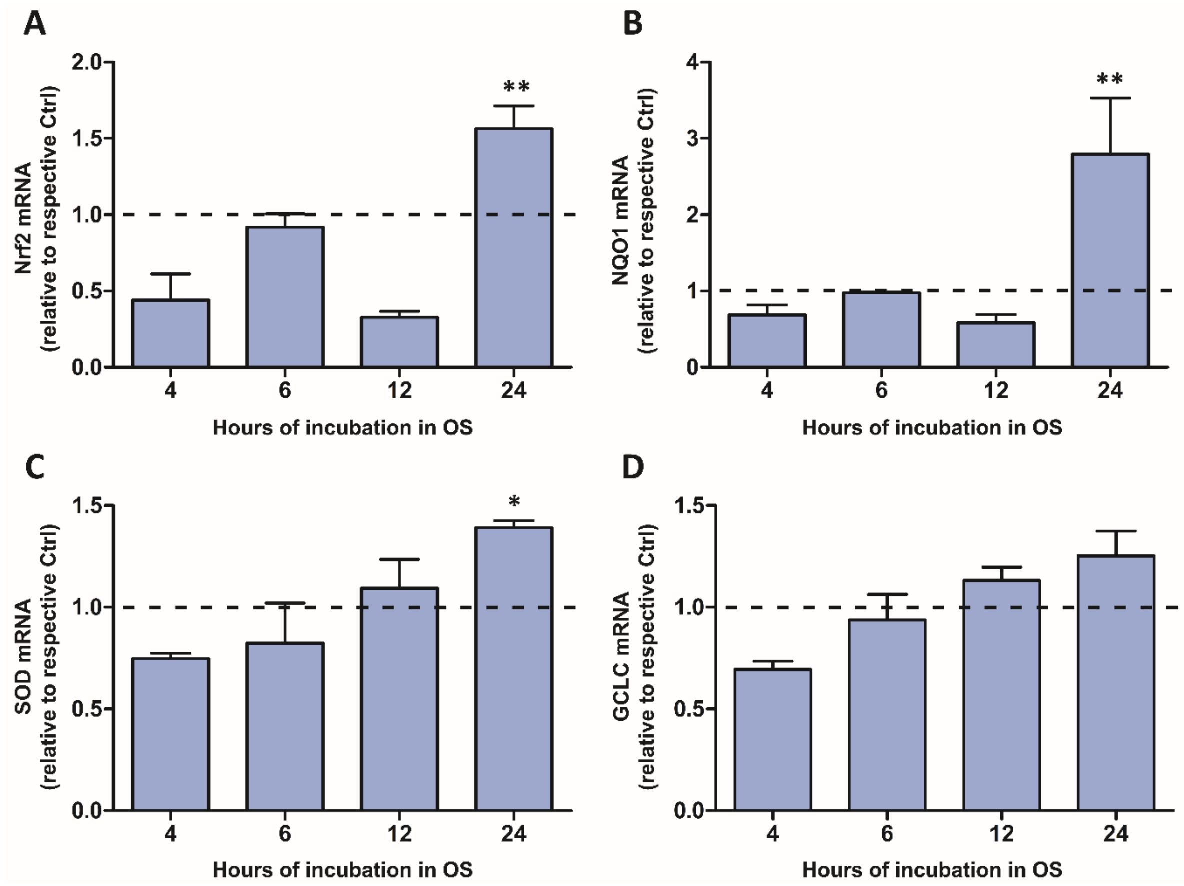

Figure S3. Time course of *Nrf2* and of oxidative stress marker expressions in response to OS to determine the shortest incubation time for detecting an effect of OS in retinal explants. * $p < 0.05$, and ** $p < 0.01$ relative to the respective control, represented by the dashed line. Two-way ANOVA followed by Bonferroni post-hoc test. $n = 3$.
